# Effects of butyric acid glycerol ester supplementation on intestinal nutrient transporter and immune-related genes in broiler chickens challenged with *Eimeria maxima*

**DOI:** 10.3389/fvets.2024.1501286

**Published:** 2025-01-22

**Authors:** Kouassi R. Kpodo, Katarzyna B. Miska, Lori L. Schreier, Daniel J. Milliken, Monika Proszkowiec-Weglarz

**Affiliations:** Animal Biosciences and Biotechnology Laboratory, Agricultural Research Service, United States Department of Agriculture, Beltsville, MD, United States

**Keywords:** butyric acid, coccidiosis, nutrient transporter genes, immune genes, chickens

## Abstract

**Introduction:**

Coccidiosis negatively affects intestinal health and digestive functions; however, whether butyric acid glycerol ester (BE) can mitigate these negative effects in broiler chickens is unknown. The study objective was to determine the effects of BE on nutrient transporter and intestinal immune genes in chickens infected with *Eimeria maxima* (EM).

**Methods:**

Ross male 708 chicks were fed diets supplemented with 0 (control, C) or 0.25% of BE. On day 21, half the chickens from each feeding group were infected with 0 or 10^3^ EM sporulated oocysts creating four treatment groups (C, +EM, +BE, and BE + EM; *n* = 6/treatment group). Jejunal and ileal tissues were collected at days 7 and 10 post-infection (PI).

**Results:**

EM infection reduced (*P* ≤ 0.02) nutrient transporter genes *EAAT3, PEPT2, B*°*AT, GLUT2, GLUT5*, and *SGLT1* at days 7 PI in the jejunum and ileum and *EAAT3, PEPT1, PEPT2*, and *B*°*AT* at day 10 PI in the jejunum. The supplementation of BE increased *CAT1* in the jejunum and *PEPT1, GLUT2*, and *GLUT5* (*P* ≤ 0.04) in the ileum at day 10 PI. A BE x EM interaction was observed (*P* ≤ 0.02) where *GLUT1* and *GLUT2* were increased in the jejunum of +BE compared to C chickens at day 10 PI. Among the immune-related genes, EM reduced (*P* ≤ 0.0001) *IgA* in the jejunum but increased (*P* = 0.004) *TGF-*β*4* in the jejunum and ileum at day 7 PI. The expression of *pIgR* was reduced, while *TLR2* and *TLR4* were increased in +EM compared to C chickens at day 7 PI. In addition, *IgA* was increased (*P* = 0.01) in the ileum of +BE compared to C chickens at day 10 PI.

**Conclusion:**

The results of the study confirmed that *Eimeria maxima* reduced nutrient transporters and immune-related genes in the jejunum and ileum of chickens. However, although BE increased the expression of some genes in non-challenged chickens, its supplementation did not prevent the reduction in the expression of selected genes caused by EM infection.

## 1 Introduction

Coccidiosis is one of the main problems facing the poultry industry and causes an estimated $13.2 billion loss annually worldwide (1). These economic losses stem from reduced weight gain and increased anticoccidial prophylaxis and treatment costs, morbidity, and mortality (1). Coccidiosis is caused by members of the genus Eimeria and seven recognized species among which *Eimeria maxima* (EM), *E. tenella, E. acervulina, E. necatrix*, and *E. brunetti* are the most pathogenic ones in chickens (2). These *Eimeria* species are protozoan parasites that invade epithelial cells of different intestinal segments where they undergo several rounds of asexual reproduction followed by sexual stages to form oocysts that are released into the intestinal lumen. These oocysts do not complete their differentiation or sporulation until a few days after they have been shed in the excreta. This replication process physically damages epithelial cells (3–5) disrupting their junctional complexes and increasing intestinal permeability (6, 7). In addition, the negative effects of the parasites on intestinal epithelium increased inflammatory responses (8, 9) and reduced nutrient digestibility and utilization (10, 11). EM infection has been shown to reduce the expression of nutrient transporter genes (12–14). Traditionally, coccidiosis has been controlled using management practices, vaccination, and anticoccidial drugs. However, because the widespread use of anticoccidial drugs has led to the development of resistance to these drugs, feed additives including organic acids such as butyrate are promoted.

Butyrate is a short-chain fatty acid that serves as an energy source for colonocytes and is involved in intestinal epithelial cell proliferation and differentiation (15). It is produced by bacterial fermentation of mainly undigested dietary carbohydrates (15) and has many beneficial effects. Butyrate increased ileal energy digestibility, villus height in the duodenum of *Salmonella*-challenged chickens (16), and amino acid digestion in chickens (17). Therefore, it has the potential to reduce coccidiosis-induced intestinal damage and increase nutrient digestibility. In addition, butyrate moderately reduced the increase of pro-inflammatory cytokines in lipopolysaccharide-injected chickens, suggesting that butyrate may help control inflammatory responses in the case of *Eimeria*-induced intestinal damage and subsequent immune response to bacterial infection (18). Although studies have investigated the effects of butyrate in chickens, limited data exist on the effects of butyrate on intestinal absorptive and immune responses during a coccidia challenge. Previous data in our laboratory have shown that butyric acid glycerol ester improved weight gain and feed conversion ratio and had a limited effect on gut microbiota in chickens challenged with EM (19, 20). The use of butyrate is challenging because parts of the dietary butyrate or butyric acid can be absorbed in the foregut before reaching the small intestine. In addition, butyrate has a repulsive odor that limits its consumption by chickens; therefore, butyrate was provided as butyric acid glycerol ester to overcome these two limitations for targeted release by lipases in the small intestine (21) making it more palatable and unlikely to reduce feed consumption (19, 22). Therefore, the study objective was to determine the effects of butyric acid glycerol ester, on nutrient absorption and intestinal immune-related genes of broiler chickens following EM infection. This investigation is part of a larger study, in which data on performance parameters, intestinal gene expression, and ileal and cecal microbiota have been published (19, 20). We hypothesized that butyric acid glycerol ester would reduce the negative impacts of EM infection on intestinal transporter and immune-related genes.

## 2 Materials and methods

### 2.1 Animal and experimental protocols

All animal procedures used in this experiment were approved by the Animal Care and Use Committee of the Beltsville Animal Agricultural Research Center (BARC), in Beltsville, Maryland (#16-018). The animal experimental protocol was previously described (19). In brief, day-old-Ross 708 male chicks purchased from Longenecker's Hatchery (Elizabethtown, PA) were raised on floor pens (four pens, 25 chickens per pen) until day 18 post-hatch. Half the chickens were fed a corn–soybean meal-based diet formulated to meet or exceed the NRC (23) requirements for broiler chickens and supplemented with 0 (control, C) or 0.25% (w/w) of butyric acid glycerol ester (BE, ProPhorce SR 130, Perstorp, Waspik, Netherlands) until the end of the experiment on day 31 post-hatch. This product allows a targeted butyric acid delivery in the intestine. At 19 day post-hatch, chickens were transferred to battery cages and allowed a 3-day acclimation period. On day 21, half the chickens from each feeding group (C and BE) were sham inoculated with 0 or infected with 10^3^ EM sporulated oocysts (24) by oral gavage creating four treatment groups (C, +BE, +EM, and BE + EM; *n* = 6 per treatment group). The BE level of 0.25% (w/w) was recommended by the manufacturer. Two chickens died before the EM infection and two after the infection (from infected and uninfected groups). Chickens (*n* = 6 per treatment group) were euthanized at days 7 and 10 post-infection (PI). EM has maximum effects on performance at days 7; therefore, we have chosen to access the gene expressions at days 7 and 10 PI, the height of the infection, and the recovery period, respectively. For each euthanasia timepoint, chickens were weighed and sampled separately. After euthanasia by cervical dislocation, 2–3 cm in the middle of the distal part of the ileum and the jejunum were excised, cleaned of digesta, snap-frozen in liquid nitrogen, and stored at −80°C for later processing for RNA extraction and reverse transcription-quantitative polymerase chain reaction (RT-qPCR).

### 2.2 RNA extraction and reverse transcription-quantitative PCR

Total RNA was extracted from intestinal tissue using the RNeasy Mini QIAcube kit and QIAcube instrument (Qiagen, Valencia, CA, USA) following the manufacturer's protocols. The quality of the resulting RNA was evaluated on a NanoDrop One (Thermo Fisher Scientific, Inc., Waltham, MA, USA), and 0.5 μg of the RNA was reverse-transcribed to cDNA using the Superscript IV reverse transcriptase (Invitrogen, Carlsbad, CA, USA) and oligo dT primers following the manufacturer's instructions. The cDNA was diluted 1:10 and used in the real-time PCR reactions, which were performed in 15 μL total volume containing 2 μL of cDNA, 300 or 400 nM of reverse and forward of each gene-specific primer, and SsoAdvanced Universal SYBR Green Supermix (Bio-Rad, Hercules, CA, USA) using the CFX96TM Touch System (Bio-Rad). A temperature gradient was performed for each primer set, and a gel was run to ensure that the primer was specific for the gene. Primer sequences were previously published and are presented in Table 1. The thermal cycling parameters were set at 95°C for 5 min followed by 40 cycles of 95°C for 15 s, 60°C for 30 s, and 72°C for 30 s. A melt curve analysis was performed at the end at 95°C, and gel electrophoresis was run on the qPCR products to ensure that the amplicon was the appropriate size and only the genes of interest were amplified. The genes investigated were Na^+^-independent cationic amino acid transporter (*CAT1*), excitatory amino acid transporter 3, Na^+^, H^+^, K^+^ dependent (*EAAT3*), oligopeptide transporter (*PEPT1*), H^+^/peptide cotransporter (*PEPT2*), Na+-dependent neutral amino acid transporter (*B*°*AT*), glucose transporter-1 (*GLUT1*), Na^+^-independent glucose, galactose, and fructose transporter (*GLUT2*), Na^+^-independent fructose transporter (*GLUT5*), Na^+^-dependent glucose and galactose transporter (*SGLT1*), immunoglobulin A (*IgA*), polymeric immunoglobulin receptor (*pIgR*), Toll-like receptors (*TLR*)*2, TLR4*, transforming growth factor 4 beta (*TGF-4*β), and tumor necrosis factor alpha (*TNF-*α). The gene expression data were normalized to the geometric mean of the three reference genes glyceraldehyde-3-phosphate dehydrogenase (*GAPDH*), beta-actin (*B-actin*), and beta-2 microglobulin (*B2-M*), and relative gene expression was calculated using the 2^−Δ*ΔCt*^ method (25).

**Table 1:** Gene-specific primers used for the analysis of mRNA levels using quantitative real-time RT-qPCR.

**Gene^a^**	**GenBank accession no**.	**Forward primer (5^′^ → 3^′^)**	**Reverse Primer (5^′^ → 3^′^)**	**Amplicon size (bp)**	**References**
B°AT	XM_419056	GGGTTTTGTGTTGGCTTAGGAA	TCCATGGCTCTGGCAGAGAT	60	^b^
B2-m	Z48921	TGGAGCACGAGACCCTGAAG	TTTGCCGTCATACCCAGAAGT	161	^b^
B-actin	X0082	TTCTTTTGGCGCTTGACTCA	TTTGCCGTCATACCCAGAAGT	88	(47)
CAT 1	NM_001145490.2	ACAGTGACATCAGGAGCCGT	AGGCTGCCACCAACGAGTAA	112	^b^
EAAT3	XM_424930.8	GCAGCACCCCAGAAGTCAGT	CCACACCGGTTGTAGCTTCC	135	^b^
GAPDH	NM_204305	AGCCATTCCTCCACCTTTGAT	AGTCCACAACACGGTTGCTGTAT	112	(47)
GLUT1	NM_205209.2	ACTTCATTGTGGGCATGGGC	CTCCGGCACCTTGAAGTAGGT	119	(47)
GLUT2	NM_207178.2	GCAACCATTGGCGTTGGAGT	ATGCCCATCAGACCAGCCAG	107	(47)
GLUT5	XR_005855627.2	TGCTATTGGAGCCAGTCCGA	AGGAACACGAGTCCCACAGT	132	^b^
IgA	S40610	GAAGGTCTCCGTGGAGGATT	ACGTTGACGTGAGAGGC	129	(48)
Pept1	NM_204365.2	GCTGCCCTCATGGCTGTTTC	CTCTTTGCTGCGATGCCGAA	147	(47)
Pept2	XM_004935370.5	TGTCCAAGAAGCAGACTTCCCA	GCAGTGTTTGGGCTTCCGAC	150	(47)
pIgR	ENSGALT00000001353	CAAGGGAGTACGGAGCAAAC	CTTTGTCTCAGCGGTGCTTT	116	(48)
SGLT1	NM_001293240.2	GCCATCGTTATCCTGGCAGTC	GCAAAGACCAGCACAAGCGA	79	(47)
TGF-β	NM_001318450	CGACCTCGACACCGACTACT	CCACTTCCACTGCAGATCCT	135	(49)
TLR4	NM_001030693	TTCCTGCTGAAATCCCAAAC	TATGGATGTGGCACCTTGAA	132	(49)
TLR2	NM_204278	TCACAGGCAAAATCACGGTG	GATTTGGTTGGACTGCAGCA	116	(49)
TNFα	NM_204267	AGTTGCCCTTCCTGTAACCA	AGAGCATCAACGCAAAAGGG	140	^b^

^a^Abbreviations of the gene names are defined in text.

^b^Primers were designed using primer3 program (http://bioinfo.ut.ee/primer3-0.4.0/primer3/).

### 2.3 Statistical analysis

Data were calculated as a percent relative to C and analyzed separately for days 7 and 10 PI with BE, EM, and BE × EM interaction as fixed effects using the GLIMMIX procedure (SAS 9.4, Cary, NC, USA). All the data were log-transformed to meet assumptions of normality and the homogeneity of variance, and back-transformed least squares means were reported. Means were separated using Tukey's adjustment, and a statistical significance was set at a *p*-value of ≤ 0.05.

## 3 Results

### 3.1 Nutrient transporter genes

#### 3.1.1 Jejunum

At 7 day PI, no significant effects of BE or BE x EM interaction were observed (*P* > 0.05) for any of the genes. However, mRNA expression of *CAT1* and *GLUT1* was increased (*P* = 0.01 and *P* = 0.02, respectively) by EM infection (Table 2). At day 7 PI, mRNA expression of *EAAT3, PEPT2, B*°*AT, GLUT2, GLUT5*, and *SGLT1* was reduced (all *P*-values < 0.0001) by EM infection (Table 2).

**Table 2:** Effects of in-feed butyric acid glycerol ester (BE) on amino acid and sugar transporter genes in the jejunum of broilers at day 7 post-*Eimeria maxima* (EM) infection.

**Treatments**									
**BE** × **EM**	**CAT1**	**EAAT3**	**PEPT1**	**PEPT2**	**B**°**AT**	**GLUT1**	**GLUT2**	**GLUT5**	**SGLT1**
C	100	98.48	100	100	100	100	100	100	100
+EM	120.1	21.67	70.79	23.7	41.85	136.23	32.83	43.01	33.58
+BE	82.13	114.78	102.99	87.96	111.45	104.4	94.07	105.06	86.78
BE + EM	131.96	22.13	79.26	29.89	38.27	137.56	33.93	29.76	7.65
**Main effects**									
**BE (% w/w)**									
0	110.05	60.08	85.39	61.85	70.93	118.12	66.42	71.51	66.79
0.25	107.05	68.46	91.13	58.92	74.86	120.98	64	67.41	47.22
**EM (oocyst)**									
0	100.74^b^	106.63^a^	101.5	93.98^a^	105.73^a^	102.20^b^	97.04^a^	102.53^a^	93.39^a^
10^3^	126.03^a^	21.90^b^	75.03	26.80^b^	40.06^b^	136.90^a^	33.38^b^	36.39^b^	20.62^b^
**Pooled SEM**	12.36	9.95	13.11	8.84	10.09	13.55	7.65	9.96	7.15
***P*** **-values**									
BE	0.81	0.57	0.67	0.74	0.7	0.84	0.76	0.69	0.56
EM	0.01	< 0.0001	0.06	< 0.0001	< 0.0001	0.02	< 0.0001	< 0.0001	< 0.0001
BE x EM	0.24	0.67	0.84	0.32	0.47	0.91	0.65	0.37	0.23

^a, b^Means with different superscripts within a column are significantly different (*P* ≤ 0.05).

CAT1, Na^+^-independent cationic amino acid transporter; EAAT3, excitatory amino acid transporter 3, Na^+^, H^+^, K^+^ dependent; PEPT1, oligopeptide transporter; PEPT2, H^+^/peptide cotransporter; B°AT, Na^+^-dependent neutral amino acid transporter; GLUT1, glucose transporter-1; GLUT2, Na^+^-independent glucose, galactose, and fructose transporter; GLUT5, Na^+^-independent fructose transporter; SGLT1, Na^+^-dependent glucose, galactose transporter; C, control; sham inoculated eating normal feed.

At 10 day PI, no BE x EM interaction was observed (*P* > 0.05) for *CAT1, EAAT3, PEPT1, PEPT2, B*°*AT, GLUT5*, and *SGLT1* mRNA expression. However, mRNA expression of *CAT1, GLUT1*, and *GLUT2* was increased (*P* = 0.01, *P* = 0.004, and *P* = 0.003, respectively) by BE, while the mRNA expression of *EAAT3, PEPT1, PEPT2, B*°*AT, GLUT2*, and *GLUT5* was reduced (*P* = 0.01, *P* = 0.004, *P* = 0.05, *P* = 0.02, *P* = 0.0004, and *P* = 0.0006, respectively) by EM (Table 3). An interaction was observed (*P* = 0.02) for *GLUT1* mRNA expression where it was increased in +BE compared to C chickens, while no differences were observed between both +EM and BE + EM compared to +BE or C chickens (Table 3). An interaction was also observed (*P* < 0.001) for *GLUT2* mRNA expression where it was increased in +BE compared to C, +EM, and BE + EM chickens (Table 3). There were no main effects of BE and EM or their interaction (*P* > 0.05) on *SGLT1* mRNA expression (Table 3).

**Table 3:** Effects of in-feed butyric acid glycerol ester (BE) on amino acid and sugar transporter genes in the jejunum of broilers at day 10 post-*Eimeria maxima* (EM) infection.

**Treatments**									
**BE** × **EM**	**CAT1**	**EAAT3**	**PEPT1**	**PEPT2**	**B**°**AT**	**GLUT1**	**GLUT2**	**GLUT5**	**SGLT1**
C	100.00	100.00	100.00	100.00	100.00	100.00^b^	100.00^b^	100.00	100.00
+EM	133.91	76.74	73.12	94.99	69.28	123.40^ab^	98.35^b^	63.00	111.26
+BE	209.56	147.79	143.64	110.30	167.46	167.80^a^	212.43^a^	108.16	103.11
BE + EM	178.43	76.18	55.73	39.50	79.08	132.37^ab^	85.82^b^	46.14	50.17
**Main effects**									
**BE (% w/w)**									
0	116.96^b^	88.37	86.56	97.50	84.64	111.70^b^	99.18^b^	81.50	105.63
0.25	194.00^a^	111.99	99.69	74.90	123.27	150.09^a^	149.13^a^	77.15	76.64
**EM (oocysts)**									
0	154.78	123.90^a^	121.82^a^	105.15^a^	133.73^a^	133.90	156.22^a^	104.08^a^	101.56
10^3^	156.17	76.46^b^	64.43^b^	67.25^b^	74.18^b^	127.89	92.09^b^	54.57^b^	80.72
**Pooled SEM**	24.77	17.06	17.74	18.26	24.10	11.50	14.62	11.99	19.38
***P*** **-values**									
BE	0.01	0.19	0.47	0.23	0.13	0.004	0.003	0.72	0.15
EM	0.96	0.01	0.004	0.05	0.02	0.61	0.0004	0.0006	0.30
BE × EM	0.21	0.18	0.10	0.09	0.25	0.02	< 0.001	0.31	0.12

^a, b^Means with different superscripts within a column are significantly different (*P* ≤ 0.05).

CAT1, Na^+^-independent cationic amino acid transporter; EAAT3, excitatory amino acid transporter 3, Na^+^, H^+^, K^+^ dependent; PEPT1, oligopeptide transporter; PEPT2, H^+^/peptide cotransporter; B°AT, Na^+^-dependent neutral amino acid transporter; GLUT1, glucose transporter-1; GLUT2, Na^+^-independent glucose, galactose, and fructose transporter; GLUT5, Na^+^-independent fructose transporter; SGLT1, Na^+^-dependent glucose, galactose transporter; C, control; sham inoculated eating normal feed.

#### 3.1.2 Ileum

At 7 day PI, no significant effects of BE or BE x EM interaction were observed (*P* > 0.05) for any of the genes. The mRNA expression of *CAT1* was increased (*P* = 0.01) by EM infection (Table 4). The mRNA expression of *EAAT3, PEPT2, B*°*AT, GLUT2, GLUT5*, and *SGLT1* was reduced (*P* < 0.0001, *P* < 0.0001, *P* < 0.0001, *P* = 0.0008, *P* = 0.0003, and *P* < 0.0001, respectively) by EM infection (Table 2). There were no main effects of EM (*P* > 0.05) for *PEPT1* and *GLUT1* mRNA expression (Table 4).

**Table 4:** Effects of in-feed butyric acid glycerol ester (BE) on amino acid and sugar transporter genes in the ileum of broilers at day 7 post-*Eimeria maxima* (EM) infection.

**Treatments**									
**BE** × **EM**	**CAT1**	**EAAT3**	**PEPT1**	**PEPT2**	**B**°**AT**	**GLUT1**	**GLUT2**	**GLUT5**	**SGLT1**
C	97.37	92.81	100.00	100.00	100.00	99.79	100.00	100.00	100.00
+EM	112.77	17.91	50.85	24.61	27.39	126.21	33.79	66.25	45.16
+BE	67.72	81.86	71.21	88.96	64.43	100.65	112.62	122.97	98.36
BE + EM	111.58	18.10	75.26	28.81	28.67	114.90	62.41	59.14	54.59
**Main effects**									
**BE (% w/w)**									
0	105.07	55.36	75.43	62.30	63.69	113.00	66.90	83.12	72.58
0.25	89.65	49.98	73.24	58.89	46.55	107.78	87.51	91.06	76.48
**EM (oocysts)**									
0	92.62^b^	87.33^a^	85.61	94.48^a^	82.22^a^	100.22	106.31^a^	111.49^a^	99.18^a^
10^3^	112.17^a^	18.01^b^	63.06	26.71^b^	28.03^b^	120.56	48.10^b^	62.69^b^	49.88^b^
**Pooled SEM**	10.86	10.70	14.47	11.26	9.87	11.29	14.69	10.65	8.18
***P*** **-values**									
BE	0.11	0.78	0.88	0.77	0.10	0.68	0.18	1.00	0.64
EM	0.01	< 0.0001	0.14	< 0.0001	< 0.0001	0.09	0.0008	0.0003	< 0.0001
BE × EM	0.13	0.74	0.08	0.51	0.08	0.62	0.59	0.72	0.51

^a, b^Means with different superscripts within a column are significantly different (*P* ≤ 0.05).

CAT1, Na^+^-independent cationic amino acid transporter; EAAT3, excitatory amino acid transporter 3, Na^+^, H^+^, K^+^ dependent; PEPT1, oligopeptide transporter; PEPT2, H^+^/peptide cotransporter; B°AT, Na^+^-dependent neutral amino acid transporter; GLUT1, glucose transporter-1; GLUT2, Na^+^-independent glucose, galactose, and fructose transporter; GLUT5, Na^+^-independent fructose transporter; SGLT1, Na^+^-dependent glucose, galactose transporter; C, control; sham inoculated eating normal feed.

At 10 day PI, the mRNA expression of *PEPT1, GLUT2*, and *GLUT5* was increased (*P* = 0.01, *P* = 0.02, and *P* = 0.04, respectively) by BE (Table 5). No other gene expression differences were observed (*P* > 0.07; Table 5).

**Table 5:** Effects of in-feed butyric acid glycerol ester (BE) on amino acid and sugar transporter genes in the ileum of broilers at day 10 post-*Eimeria maxima* (EM) infection.

**Treatments**									
**BE** × **EM**	**CAT1**	**EAAT3**	**PEPT1**	**PEPT2**	**B**°**AT**	**GLUT1**	**GLUT2**	**GLUT5**	**SGLT1**
C	97.29	100.00	100.00	100.00	100.00	98.62	100.00	100.00	100.00
+EM	95.41	84.70	98.02	84.85	83.45	90.01	84.69	75.03	108.03
+BE	92.14	102.38	60.56	94.98	115.36	94.99	67.86	71.81	104.46
BE + EM	97.49	72.10	52.61	64.11	73.37	113.14	48.39	53.29	83.25
**Main effects**									
**BE (% w/w)**									
0	96.35	92.35	99.01^a^	92.42	91.73	94.32	92.35^a^	87.51^a^	104.02
0.25	94.82	87.24	56.58^b^	79.55	94.37	104.07	58.13^b^	62.55^b^	93.86
**EM (oocysts)**									
0	94.71	101.19	80.28	97.49	78.41	96.81	83.93	85.91	102.23
10^3^	96.45	78.40	75.32	74.48	107.68	101.58	66.54	64.16	95.64
**Pooled SEM**	10.64	15.20	14.10	12.73	17.28	7.13	13.32	11.29	15.51
***P*** **-values**									
BE	0.88	0.74	0.01	0.32	0.88	0.20	0.02	0.04	0.52
EM	0.87	0.15	0.73	0.09	0.11	0.57	0.21	0.07	0.68
BE × EM	0.74	0.63	0.83	0.54	0.47	0.08	0.88	0.78	0.36

^a, b^Means with different superscripts within a column are significantly different (*P* ≤ 0.05).

CAT1, Na^+^-independent cationic amino acid transporter; EAAT3, excitatory amino acid transporter 3, Na^+^, H^+^, K^+^ dependent; PEPT1, oligopeptide transporter; PEPT2, H^+^/peptide cotransporter; B°AT, Na^+^-dependent neutral amino acid transporter; GLUT1, glucose transporter-1; GLUT2, Na^+^-independent glucose, galactose, and fructose transporter; GLUT5, Na^+^-independent fructose transporter; SGLT1, Na^+^-dependent glucose, galactose transporter; C, control; sham inoculated eating normal feed.

### 3.2 Immune-related genes

#### 3.2.1 Jejunum

At 7 day PI, the mRNA expression of *IgA* and *pIgR* was reduced (*P* = 0.001 and *P* = 0.05, respectively) by EM infection (Table 6). A BE × EM interaction was observed (*P* = 0.004) for *pIgR* mRNA expression where it was reduced in +EM and +BE compared to C chickens (Table 6). The mRNA expression of *TGF-*β*4* and *TLR2* was increased (*P* < 0.0001 and *P* = 0.004, respectively) by EM infection (Table 6). A BE × EM interaction was observed (*P* = 0.015) for *TLR2* mRNA where it was increased in +EM compared to C while no differences were observed between both +BE and BE + EM compared to +EM or C chickens (Table 6). A BE × EM interaction was observed (*P* = 0.003) for *TLR4* mRNA where its expression was increased in +EM compared to C and BE + EM (Table 6). No other differences were observed for any genes (*P* > 0.19; Table 6).

**Table 6:** Effects of in-feed butyric acid glycerol ester (BE) on immune-related genes in the jejunum of broilers at day 7 post-*Eimeria maxima* (EM) infection.

**Treatments**						
**BE** × **EM**	**IgA**	**pIgR**	**TGF-**β**4**	**TLR2**	**TLR4**	**TNF-**α
C	100.00	100.00^a^	100.00	100.00^b^	100.00^b^	100.00
+EM	25.62	45.38^b^	157.03	292.09^a^	161.24^a^	86.68
+BE	78.58	53.10^b^	84.76	167.52^ab^	130.29^ab^	96.52
BE + EM	35.08	65.21^ab^	167.06	187.46^ab^	91.56^b^	96.11
**Main effects**						
**BE (% w/w)**						
0	62.81	72.69	128.52	196.05	130.62	93.34
0.25	56.83	59.15	125.91	177.49	110.92	96.31
**EM (oocysts)**						
0	89.29^a^	76.55^a^	92.38^b^	133.76^b^	115.15	98.26
10^3^	30.35^b^	55.30^b^	162.05^a^	239.78^a^	126.40	91.39
**Pooled SEM**	15.39	10.10	11.66	32.30	14.41	9.78
***P*** **-values**						
BE	0.70	0.20	0.83	0.57	0.19	0.76
EM	0.001	0.05	< 0.0001	0.004	0.45	0.49
BE × EM	0.33	0.004	0.29	0.015	0.003	0.52

^a, b^Means with different superscripts within a column are significantly different (*P* ≤ 0.05).

IgA, Immunoglobulin A; pIgR, polymeric immunoglobulin receptor; TLR- β4, Toll-like receptor beta; TLR2, Toll-like receptor; TGF-4β, transforming growth factor beta; TNF-α, tumor necrosis factor alpha; C, control; sham inoculated eating normal feed.

At 10 day PI, a BE by EM interaction was observed for *TGF-*β*4* mRNA where its expression was reduced in BE+EM compared to +EM while no differences were observed between both +BE and C compared to BE + EM or +EM chickens (Table 7). No other immune gene expression differences were observed (*P* ≥ 0.07; Table 7).

**Table 7:** Effects of in-feed butyric acid glycerol ester (BE) on immune-related genes in the jejunum of broilers at day 10 post-*Eimeria maxima* infection (EM).

**Treatments**						
**BE** × **EM**	**IgA**	**pIgR**	**TGF-B4**	**TLR2**	**TLR4**	**TNF**α
C	100.00	100.00	96.79^ab^	100.00	100.00	100.00
+EM	98.55	87.88	158.46^a^	122.01	101.98	137.69
+BE	143.81	100.67	99.04^ab^	99.10	105.17	113.43
BE + EM	119.42	82.26	92.46^b^	66.77	57.62	90.58
**Main effects**						
**BE (% w/w)**						
0	99.27	93.94	127.63	111.01	100.99	118.85
0.25	131.62	91.47	95.75	82.94	81.39	102.01
**EM (oocysts)**						
0	121.91	100.34	97.92	99.55	102.59	106.72
10^3^	108.98	85.07	125.46	94.39	79.80	114.14
**Pooled SEM**	19.34	19.19	14.78	23.13	22.51	22.05
***P*** **-values**						
BE	0.11	0.90	0.07	0.24	0.40	0.46
EM	0.51	0.44	0.13	0.83	0.33	0.74
BE × EM	0.56	0.87	0.05	0.26	0.29	0.19

^a, b^Means with different superscripts within a column are significantly different (*P* ≤ 0.05).

IgA, Immunoglobulin A; pIgR, polymeric immunoglobulin receptor; TLR- β4, Toll-like receptor beta; TLR2, Toll-like receptor; TGF-4β, transforming growth factor beta; TNF-α, tumor necrosis factor alpha; C, control; sham inoculated eating normal feed.

#### 3.2.2 Ileum

At 7 day PI, the expression of *TGF-*β mRNA was increased (*P* = 0.004) by EM infection whereas it was decreased (*P* = 0.04) by BE (Table 8). No other gene expression differences were observed (*P* ≥ 0.06; Table 8).

**Table 8:** Effects of in-feed butyric acid glycerol ester (BE) on immune-related genes in the ileum of broilers at day 7 post-*Eimeria maxima* infection (EM).

**Treatments**						
**BE** × **EM**	**IgA**	**pIgR**	**TGF-**β**4**	**TLR2**	**TLR4**	**TNF**α
C	100.00	100.00	94.41	87.11	97.31	100.00
+EM	89.92	88.78	138.15	128.97	104.09	96.53
+BE	135.34	92.74	77.01	85.76	91.58	107.61
BE + EM	59.26	87.48	106.14	57.63	77.27	97.64
**Main effects**						
**BE (% w/w)**						
0	94.96	94.39	116.28^a^	108.04	100.70	98.27
0.25	97.30	90.11	91.57^b^	71.69	84.42	102.62
**EM (oocysts)**						
0	117.67	96.37	85.71^b^	86.43	94.44	103.81
10^3^	74.59	88.13	122.14^a^	93.30	90.68	97.09
**Pooled SEM**	20.85	12.94	11.20	21.84	15.94	6.24
***P*** **-values**						
BE	0.91	0.74	0.04	0.11	0.31	0.49
EM	0.06	0.53	0.004	0.99	0.77	0.30
BE × EM	0.13	0.82	0.79	0.12	0.50	0.61

^a, b^Means with different superscripts within a column are significantly different (*P* ≤ 0.05).

IgA, Immunoglobulin A; pIgR, polymeric immunoglobulin receptor; TLR- β4, Toll-like receptor beta; TLR2, Toll-like receptor; TGF-4β, transforming growth factor beta; TNF-α, tumor necrosis factor alpha; C, control; sham inoculated eating normal feed.

At 10 day PI, a BE x EM interaction was observed for *IgA* mRNA where its expression was increased in +BE compared to C chickens while no differences were observed between both +EM and BE + EM compared to +BE or C chickens (Table 9). No other gene expression differences were observed (*P* ≥ 0.06; Table 9).

**Table 9:** Effects of in-feed butyric acid glycerol ester (BE) on immune-related genes in the ileum of broilers at day 10 post-*Eimeria maxima* infection (EM).

**Treatments**						
**BE** × **EM**	**IgA**	**pIgR**	**TGF-**β**4**	**TLR2**	**TLR4**	**TNF**α
C	100.00^b^	100.00	100.00	100.00	97.14	100.00
+EM	162.27^ab^	86.81	125.33	153.96	102.11	85.55
+BE	220.11^a^	72.62	133.29	108.81	100.85	86.12
BE + EM	125.42^ab^	93.13	133.10	135.57	107.65	99.82
**Main effects**						
**BE (% w/w)**						
0	131.14	93.40	112.67	126.98	99.63	92.77
0.25	172.77	82.88	133.20	122.19	104.25	92.97
**EM (oocysts)**						
0	160.06	86.31	116.65	104.41	99.00	93.06
10^3^	143.85	89.97	129.22	144.77	104.88	92.68
**Pooled SEM**	26.91	9.38	17.41	23.95	23.48	7.11
***P*** **-values**						
BE	0.14	0.28	0.25	0.84	0.85	0.98
EM	0.55	0.70	0.48	0.11	0.81	0.96
BE × EM	0.01	0.09	0.47	0.58	0.97	0.06

^a, b^Means with different superscripts within a column are sig nificantly different (*P* ≤ 0.05).

IgA, Immunoglobulin A; pIgR, polymeric immunoglobulin receptor; TLR- β4, Toll-like receptor beta; TLR2, Toll-like receptor; TGF-4β, transforming growth factor beta; TNF-α, tumor necrosis factor alpha; C, control; sham inoculated eating normal feed.

## 4 Discussion

The expression of nutrient transporter genes can be reduced by EM (7, 26) increasing the necessity to find strategies to mitigate these negative impacts. In the current study, chickens were fed a BE-supplemented diet for 3 weeks prior to EM infection to determine BE effects on the negative impacts of EM on nutrient transporters and immune-related genes. EM infection decreased mRNA expression of basolateral *B*°*AT* and *EAAT3* at days 7 and 10 in the jejunum but only at day 7 PI in the ileum. The reduction in *B*°*AT* expression level has been previously reported at 6 day PI when chickens were challenged with EM, *E. acervulina*, and *E. tenella* (31) and suggests that *Eimeria* infection may have reduced the absorption of neutral amino acids. The transporter *EAAT3* is responsible for the uptake of anionic amino acids including Asp, Gln, Glu, and Cys. Among these amino acids, Glu serves as a primary energy source for the enterocytes and stimulates cellular proliferation (27, 28). The downregulation of *EAAT3* in the jejunum and ileum suggests a reduction in amino acid transport likely affecting the amino acid requirements for broiler chickens during EM and the PI period. Although the intestinal morphology was not assessed in the current study, it is well-established that EM infection physically damages intestinal epithelial cells, and the decreased expression of this amino acid transporter gene is likely to delay the restoration of epithelial cell integrity and have overall negative effects on nutrient utilization and subsequent weight gain as previously reported at 7 day PI (13, 19) and 6 day PI (26). In addition, the negative effects of EM could persist long after the initial infection since most of the genes were reduced at day 10 PI in the jejunum.

Another basolateral amino acid transporter *CAT1* involved in the uptake of cationic amino acids including Lys, Arg, and His from enterocytes into the bloodstream (29) was upregulated during EM infection in both the jejunum and the ileum at day 7 PI but not at day 10 in the current study. These results agree with those reported in a previous study where *CAT1* was upregulated at day 6 PI in the jejunum of EM-infected chickens (26). The upregulation of *CAT1* may be partially explained by the fact that the luminal absorption of amino acids was reduced due to EM and the increase in basolateral amino acid transporters was a mechanism to uptake more amino acids into the bloodstream. Alternatively, the increase in *CAT1* may be a mechanism to deplete enterocytes of nutrients and accelerate apoptosis to fight EM infection as previously hypothesized (30).

Protein digestion also yields di- and tripeptides, which are transported and further hydrolyzed in the enterocytes or absorbed into the general circulation (29). The transporters PEPT2 and PEPT1 are responsible for the uptake of di- and tripeptides (31). The reduction of the *PEPT2* mRNA suggests that the absorption of small peptides and their hydrolysis in the enterocytes may be hindered during EM infection. Contrary to *PEPT2, PEPT1* was not affected at day 7 but at day 10 PI only jejunum (31). The results of the current study are supported by a previous report in which *PEPT1* expression level was not affected at the peak of infection when chickens were infected at day 14 post-hatch (14). In another study where pullets were co-infected at day 16 post-hatch with *E. acervulina* and *E. tenella, PEPT1* decreased at day 6 PI (6). These discrepancies could be explained by the type (single *Eimeria* sp. vs. co-infection with multiple *Eimeria* sp.) and the timing of the infection. Although the reason why *PEPT1* was only affected at day 10 PI in the current study is unclear, we observed some numerical reduction of *PEPT1* mRNA level at day 7 PI, and the effect was more apparent at day 10 PI. Day 7 PI corresponds to the peak of EM infection (32) suggesting maximum epithelial damage. It generally takes 3–5 days for a complete epithelial cell turnover (33), and day 10 PI may have not been enough for complete intestinal tissue repairs. Alternatively, a secondary EM oocyst load may have occurred (32) increasing the damage and reducing the expression of *PEPT1* gene in the jejunum at day 10 PI and subsequent transport of small peptides into the enterocytes.

In addition to amino acid and peptide transporters, sugar transporters *GLUT1, GLUT2, GLUT5*, and *SGLT1* were affected by EM infection. The glucose transporter GLUT1 is a facilitated glucose transporter of low glucose levels and is ubiquitously expressed in several organs throughout the body, including the intestine (34). The upregulation of *GLUT1* at day 7 in the jejunum by EM could be an adaptation to increase the uptake of glucose, which may be most needed at the peak of EM infection when the immune system activation may be maximal since the immune system is an obligated glucose utilizer (35). However, another facilitated glucose transporter *GLUT2* located at the basolateral membrane was downregulated in the jejunum at days 7 and 10 PI but only at day 7 PI in the ileum. These results are consistent with previous reports where *GLUT2* was decreased by EM in the jejunum of broiler chickens (13). The contradiction between the expression levels of *GLUT1* and *GLUT2* suggests a more complex mechanism for the regulation of facilitated glucose uptake during EM infection. Low glucose concentration is actively absorbed from the intestinal lumen into the enterocytes via SGLT1 (36). The downregulation of *SGLT1* in the current study suggests that the glucose absorption may have been compromised and this could reduce the growth rate of EM-infected chickens as previously reported (19). The glucose transporter GLUT5, a luminal fructose transporter into the enterocytes (37), was also reduced by EM infection agreeing with previous reports (13, 38). The downregulation of *GLUT5* and *GLUT2* (also involved in fructose transport) in the current study suggests that fructose absorption into the enterocytes and the bloodstream may have been compromised by EM infection.

In general, EM infection reduced most of the nutrient transporter genes in both the jejunum and the ileum, but the BE supplementation did not prevent these negative effects of EM infection. Among the di- and tripeptides, amino acids, and sugar transporters, we observed EM x BE interaction only for *GLUT1* and *GLUT2* in the jejunum at day 10 PI. The expression level of *GLUT1* was increased in +BE compared to C chickens, and *GLUT2* was increased in +BE compared to the rest of the treatment groups; however, the interactions were mainly due to the presence of BE in non-infected chickens. In addition, the main effect of BE reduced the expression of *GLUT2* and *GLUT5* in the ileum at day 10 PI regardless of infections. These results are perplexing because BE alone increased the expression of nutrient transporter genes but was not able to reduce chickens subjected to EM infection. However, BE supplementation improved production performance as previously reported in the companion paper (19), but perhaps not through the mediation of these genes. More research should be conducted to better understand the interaction between BE and EM as well as the effects of BE supplementation alone on nutrient transporter genes in broiler chickens without EM infection.

*Eimeria* infection physically damages intestinal mucosa (9) increasing nutrients need not only for the repair of intestinal epithelial cells but also for immune activation and maintenance (39). Immunoglobulin A is the most predominant immunoglobulin and plays an important role in the mucosal immune system (40). Its direct anticoccidial role has been reported against *E*. *tenella* through the disruption of the formation of second merozoites (41). In the current study, the gene expression of *IgA* was reduced by EM infection, suggesting a reduced mucosal immune response during the infection, especially at day 7 PI in the jejunum. Contrary to this result, the IgA protein level was increased by *E. tenella* alone or *E. tenella, E. acervulina*, and EM co-infection in the duodenum, jejunum, and ileum of broiler chickens (42). Although the IgA protein level in intestinal mucosa was not measured in the current study, it is possible that the actual protein in the intestinal mucosa of chickens in the current study may have been affected. In addition to the main effect of EM in the jejunum, *IgA* was affected by EM and BE interaction at day 10 PI in the ileum. This interaction was mainly due to BE because the differences were observed only between +BE and C chickens and BE was not effective in mitigating the negative effects of EM in the ileum. The IgA is transported into the intestinal lumen by pIgR (43). The receptor *pIgR* was affected by EM × BE interaction only at day 7 PI in the jejunum where its expression level in BE + EM chickens was intermediate to those of +EM and C chickens, suggesting that BE may have minimal effects on *pIgR* during EM infection.

Transforming growth factor, a multifunctional cytokine involved in wound healing and mucosal repair (44), was upregulated by EM infection at day 7 PI in both the jejunum and the ileum. This is expected as the intestinal mucosa of the infected chickens may have suffered physical damage (9), and *TGF-4*β was upregulated as a result to accelerate the intestinal epithelial repair. At day 10 PI, *TGF-4*β was affected by the interaction between EM and BE, and its mRNA expression was increased in +EM compared to BE + EM chickens suggesting that the supplementation of BE was not effective in the infected birds. Other immune-related genes, *TLRs*, were also affected only in the jejunum at day 7 PI. Toll-like receptors are part of the innate immune system and are involved in the first line of defense of the organism against intestinal microbes. TLR2 and TLR4 can recognize various microbial components including glycoinositol phospholipid generally present in protozoans (45) and activate the associated signaling pathways to promote the repair of injured intestinal epithelial cells and intestinal homeostasis (46). In the current study, *TLR2A* and *TLR4* increased only in +EM compared to C chickens at day 7 PI in the jejunum suggesting that they may have been upregulated to accelerate tissue repair from damage caused by EM regardless of BE supplementation. The intestinal immune system is generally affected by the presence of commensal bacteria, and one could argue that these receptors were upregulated by bacteria present in the gut; however, BE showed limited effects on the microbiota as previously reported in the companion paper of this study (20).

## 5 Conclusion

We hypothesized that BE supplementation would reduce the negative effects of EM infection on intestinal nutrient transporters and immune-related genes. Although the main effect of BE increased some nutrient transporter genes in non-challenged chickens at day 10 PI in the jejunum and the ileum and BE × EM interaction was observed for some immune-related genes at day 7 PI in the jejunum, BE supplementation was not effective in preventing the negative effects of EM infection on nutrient transporter and immune-related genes. Regardless, these data confirm that EM infection reduced most of the genes at day 7 PI in both the jejunum and the ileum and mainly at day 10 in the jejunum suggesting that the recovery from EM infection may differ depending on the intestinal segments and the time PI. These results will allow us to explore different doses of BE alone to optimize its inclusion levels or in combination with other compounds against coccidiosis in the different intestinal segments of broiler chickens.

## Author's note

Mention of trade name, proprietary product, or specific equipment does not constitute guarantee or warranty by USDA and does not imply its approval to the exclusion of other suitable products.

## Data Availability

The original contributions presented in the study are included in the article/supplementary material, further inquiries can be directed to the corresponding author/s.
